# Estimating the Prevalence of Fetal Alcohol Syndrome: A Summary

**Published:** 2001

**Authors:** Philip A. May, J. Phillip Gossage

**Affiliations:** Philip A. May, Ph.D., is a professor of sociology and senior research scientist and J. Phillip Gossage, Ph.D., is a senior research scientist at the University of New Mexico Center on Alcoholism, Substance Abuse, and Addictions (CASAA), in Albuquerque, New Mexico, where Dr. May also serves as co-director

**Keywords:** fetal alcohol syndrome, prevalence, epidemiological indicators, alcohol-related neurodevelopmental disorder, birth defects, statistical estimation, data collection, clinical aspects, population dynamics, risk factors, research in practice, research quality

## Abstract

Since the late 1970s, many studies have reported on the prevalence of fetal alcohol syndrome (FAS), alcohol-related birth defects (ARBD), and alcohol-related neurodevelopmental disorders (ARND). The three main types of research methods used in these studies are passive surveillance, clinic-based studies, and active case ascertainment. This article describes each of these methods, including their strengths and weaknesses, and summarizes the estimated prevalence of FAS produced by each of these approaches. The maternal risk factors associated with FAS and other alcohol-related anomalies include advanced maternal age, low socioeconomic status, frequent binge drinking, family and friends with drinking problems, and poor social and psychological indicators. Overall, the available literature points to a prevalence rate of FAS of 0.5 to 2 cases per 1,000 births in the United States during the 1980s and 1990s.

Establishing the prevalence^1^ and other epidemiological characteristics of fetal alcohol syndrome (FAS), alcohol-related birth defects (ARBD), and alcohol-related neurodevelopmental disorder (ARND)^2^ has been a difficult challenge ever since Jones and colleagues (Jones and Smith 1973; Jones et al. 1973) described the first cases of FAS. Researchers have been constantly challenged by issues related to case finding, sampling, diagnostic criteria, and the coordination of interdisciplinary activities. Although the diagnostic features of FAS are generally well established, the specific assessment techniques used to make the definitive diagnosis are still matters of debate. Furthermore, the criteria for ARBD and ARND (formerly referred to as fetal alcohol effects [FAE]) remain even more in question today (Stratton et al. 1996; Aase 1994; Aase et al. 1995; Astley and Clarren 2000). Because of questions of assessment methods and difficulties associated with access to cases, studies that have attempted to determine the prevalence of FAS, ARBD, and ARND are limited in number, vary widely in their methodology, and may leave the typical reader puzzled about the true pattern and the frequency of occurrence of these disorders.

This article will summarize the common methods used to study the prevalence and other epidemiological characteristics of FAS in the United States and review both similar and unique findings that have emerged in the literature from other countries. For each method, we present a survey of the studies conducted and summarize the research findings. We will highlight the biases, strengths, weaknesses, and key findings produced by each approach, and discuss the different populations studied.

## Three Common Approaches to the Epidemiological Study of FAS

Researchers used three main approaches to study the prevalence and patterns of occurrence of FAS, ARBD, and ARND: passive systems, clinic-based studies, and active case ascertainment approaches. Of these three approaches, clinic-based studies are the most common, followed by passive systems, and then active case ascertainment. Passive systems are generally the least expensive, followed by clinic-based studies; active case ascertainment studies are frequently the most costly and time intensive (Stratton et al. 1996).

### Passive Surveillance Systems

Researchers using passive systems to study FAS epidemiology use existing record collections in a particular geographical catchment area (e.g., a town or state). Researchers must first establish the criteria for defining a diagnosis of FAS, ARBD, or ARND, and then a team of reviewers looks for documented or probable cases of children born with FAS and diagnosed in a particular time period. Three types of records are generally reviewed: birth certificates, special registries for children with developmental disabilities or birth defects, and/or the medical charts of hospitals and physicians. The Centers for Disease Control and Prevention (CDC) maintains the Birth Defect Monitoring Program (BDMP) in hospitals throughout the United States, tracking most major anomalies that occur at birth, including FAS (Chavez et al. 1988). Many recent passive surveillance studies have used multiple types of records to identify as many cases of alcohol-related anomalies as possible, since a case of FAS is frequently documented in more than one place (e.g., physician records, school records, and birth certificates) over time. These multiple records approaches are referred to as capture-recapture methods (Egeland et al. 1998).

**The Use of the Terms “Prevalence” and “Incidence” in FAS Studies**

The term “prevalence” is used in this article to describe the frequency of occurrence or presence of fetal alcohol syndrome (FAS), alcohol-related birth defects (ARBD), and alcohol-related neurodevelopmental disorder (ARND) among the study population and any subgroups within the population at all time periods during the life span. In the context of FAS, some researchers (e.g., Abel and Sokol 1987, 1991; Abel 1995) use the term “incidence” to describe new FAS cases (e.g., births) each year and use the term “prevalence” to indicate the rate of FAS cases within age categories beyond birth or the first year. Without providing a detailed explanation of the conventional use of these terms in other areas of epidemiology (i.e., the way the terms are commonly used for infectious diseases), incidence means *new* cases occurring within a period of time, whereas prevalence means all *new and existing* cases during a particular time period.

The authors of this article and their colleagues have used the aforementioned terms (i.e., “incidence” and “prevalence”) in an attempt to be more consistent with gestational considerations that are important to the study of birth defects. Because FAS can exist theoretically in a fetus for up to 7 months prior to birth, the following question arises: When is FAS considered to be a new case? Because frequent spontaneous abortions occur among alcohol-abusing women, the prevalence of FAS during certain months of pregnancy may actually be much higher than the number of FAS cases recorded at birth. Because this issue poses a variety of difficult questions, the term “prevalence” is used here for all age groups. Therefore, the term “birth prevalence” is used, as is common in the study of some birth defects, rather than the term “incidence,” which is used for the onset of new cases for most other morbid conditions, especially infectious diseases. Because FAS, ARBD, and ARND are conditions that generally persist with an individual for life, “prevalence” is the term used to describe the existence of these conditions among all age groups.

—Philip A. May and J. Phillip Gossage

The major advantage of passive methods is that they efficiently utilize existing health care systems, programs, and records that are already funded by other sources. This approach is therefore relatively inexpensive and easier to undertake than some other research methods. But there are also major disadvantages. Some birth defects, such as severe spina bifida and Down’s syndrome, are easy to recognize and diagnose because the anatomical or genetic markers are obvious and well known to most obstetricians and pediatricians. FAS, ARBD, and ARND, however, are complex, involving multiple indicators of physiology, development, and behavior, many of which are not obvious at all or are at least more difficult to identify at particular ages (e.g., birth) (see Little et al. 1990; Aase 1994; Clarren et al. 2001). Therefore, passive systems, which generally depend on the diagnoses of many hundreds of non-specialist physicians, educators, and other service providers (who may miss FAS symptoms because of the circumstances of examination or the age at which the child is presented), lack the rigor and consistency of diagnoses that characterize other systems. Furthermore, passive systems depend on a variety of registries for complete and consistent records and are therefore vulnerable to the many contingencies that affect the quality of data in institutions where these data are collected.

### Clinic-Based Studies

Clinic-based studies conducted throughout the United States and the world have provided much of the current knowledge about the epidemiology of FAS and other alcohol-related disorders (Stratton et al. 1996, Abel and Sokol 1987, 1991; Abel 1995). This prospective research lends itself to a consistent design and rigorous methodology that can control for many of the problems inherent in the passive methods.

Clinic-based studies are generally conducted in prenatal clinics of large hospitals where researchers can collect data from mothers as they pass through the various months of their pregnancies. Researchers collect information from pregnant women about their diets, jobs, social interactions, psychological health, and alcohol, tobacco, and other drug use using standard screening instruments and specimen samples. Control groups are easy to obtain, since all consenting women in the clinics are screened. Generally one-half to a very substantial majority of women will report abstaining, providing an adequate comparison group. Due to the prospective nature of these designs, researchers are generally able to examine the infants at birth (and sometimes for some months postpartum) and match the maternal behaviors with the pregnancy outcomes.

Clinic-based studies have many advantages: the opportunity to gather maternal history data; the opportunity to study a large number of pregnancies with various levels of alcohol and other drug exposure; health services are provided, offering incentives for participants; and the prospective design provides greater control and rigor in measuring most of the important variables. However, there are also disadvantages. Subjects are self-selected. The women at highest risk for FAS offspring are less likely to attend prenatal clinics regularly, and many do not attend at all, making access to the very highest risk cases less regular or impossible with these methods. A second problem is that many, if not most, of the clinic-based studies conducted in the United States have been carried out in publicly funded hospitals and clinics where disadvantaged populations predominate. Therefore, clinic studies and the data obtained may overrepresent the prevalence of FAS and the characteristics of these selected populations, and underrepresent middle- and upper-class populations. Third, since FAS is not most accurately diagnosed at birth, but between the ages of 3 and 12 years, these studies may also underestimate the prevalence of FAS in the population studied (Aase 1994; Stratton et al. 1996; Clarren et al. 2001).

### Active Case Ascertainment Methods

This approach to studying FAS, like the passive surveillance method, focuses on large populations in particular geographical or catchment areas, such as schools, towns, and Indian reservations. Active case ascertainment studies are unique in that they actively seek, find, and recruit children who may have FAS within the population under study. Once researchers set the criteria for referral to clinical examination and testing, and establish a referral network and referral procedures, clinical specialists examine possible cases and assess the physical growth and development, dysmorphology, and psychosocial characteristics of the children for a final diagnosis. In some of these studies, researchers also recruit the children’s mothers to collect maternal behavior and risk factor information via interview or medical chart review.

The active case ascertainment methods have at least three advantages. One, the primary focus is on finding children with FAS at appropriate ages for accurate diagnosis by clinical specialists. Two, active, effective, and comprehensive outreach in a large general population is most likely to uncover children with FAS and alcohol-abusing mothers at the highest risk. Three, by studying entire communities or populations, this method can eliminate much selectivity and generally ensure wide representation. Therefore, an efficient active case ascertainment approach may produce the most complete access to children with FAS and the most complete assessment of the prevalence and population-based characteristics of FAS in a particular population.

There are also substantial disadvantages that can negate the benefits of this approach. First, such research is very labor intensive, time consuming, and costly (see Stratton et al. 1996). The outreach process involves gaining permission to access a community for study, training people to recognize symptoms and refer children suspected of having FAS, locating and securing permission for maternal and child subjects, hiring specialists for the clinical assessments, and holding special “developmental clinics” that may require 2 to 3 hours to completely diagnose a single child.

Second, studies of this type require cooperation from many non-researchers in the study population (e.g., community, political, health, and education officials, parents, social welfare personnel, etc.). If a vital community constituency does not support a study, case finding may be incomplete or selective, resulting in underrepresentation of the prevalence or a skewed understanding of the true characteristics of the problem. High levels of cooperation with research on stigmatized topics such as FAS and maternal drinking are often difficult to achieve.

Third, access to particular populations may be selective, and frequently only high-risk populations have been studied using these methods. In other words, these studies have been most frequently carried out where there are a large number of FAS cases to be found. If such selective populations are studied and these findings projected to the general population, then the prevalence of FAS may be overestimated.

## Prevalence Estimates by Methodology and Population

Estimates of the prevalence of FAS vary greatly from population to population and from study to study. Some of this variation is a valid reflection of the differences in FAS rates between populations, each of which may possess a number of unique risk factors, especially variations in maternal drinking behavior. But variance in rates between studies can often merely be a function of the different research methods used to study the problem. The following section reviews various studies and their findings, by method and by population (see table 1).

### Passive Systems

The CDC has published three estimates of FAS rates in the United States based on passive surveillance via the BDMP. This system uses hospital discharge data from 10 to 30 percent (depending on the year) of all births in the country. Based on this system, the estimated rate of FAS at birth was 2.0 per 10,000 (0.2 per 1,000) births from 1979 through 1992 (CDC 1993).^3^ Increased rates of 3.7 and 6.7 per 10,000 (0.37 and 0.67 per 1,000) were reported in 1992 and 1993 (CDC 1995). The researchers questioned, however, whether this increase reflected a true increase in FAS births or better reporting within the system. A third report produced from the BDMP estimated FAS rates by ethnic group from 1981 through 1986 (Chavez et al. 1988). The estimated rates per 10,000 (and per 1,000) were 6.0 (0.6) for African-Americans, 0.8 (0.08) for Hispanics, 29.9 (2.9) for American Indians, 0.3 (0.03) for Asians, and 0.9 (0.09) for whites. The rates produced by this passive system are much lower than those produced by any other method.

Local studies that have used passive methods (e.g., the Metropolitan Atlanta Congenital Defects Program and the Metropolitan Atlanta Developmental Disabilities Surveillance Program) have also produced low or modest estimates of the prevalence of FAS, especially in larger, more urban populations. In Atlanta, (CDC 1997) researchers reported the rate of FAS among newborns as 1.0 per 10,000 (0.1 per 1,000). Including “partial FAS,” the rate was 2.5 per 10,000 (0.25 per 1,000). In Alaska, a capture-recapture study used multiple sources of records, including data from hospitals, pediatricians, birth and death certificates, Alaska Native Health Service records, and genetics, disabilities, and learning programs. Researchers reported that the FAS rate for 1977–1992 for the non-Native population was 0.2–0.3 per 1,000 and that the rate for the Native population (where active case ascertainment methods were simultaneously being used by the Alaska Native Health Service) was 3.0–5.2 per 1,000 (Egeland et al. 1998). In North Dakota, Burd and colleagues (1996) reported rates of 1.1–2.0 per 1,000 based on data from the state’s birth registry system. Finally, in a recent study from New Zealand that used passive methods, pediatricians were asked to complete a postal survey designed to compile data about children with alcohol-related birth defects. For 1993, the rate of FAS among children less than ten years of age was reported as 0.11 per 1,000 (Leversha and Marks 1995).

This review of FAS rates produced by passive systems clearly indicates that very low rates of FAS are reported within the large populations studied, particularly for non-Indian and non-Native populations, and higher rates are reported for American Indians and Alaska Natives.

### Clinic-Based Studies

The clinic-based approach is the most common method used to estimate the prevalence of FAS, ARBD, and ARND, and to define maternal risk factors for these problems. A substantial number of studies of this type have been conducted. Abel and Sokol’s (1987) review of 18 clinic-based, mostly prospective studies reported an average rate of FAS for the western world of 1.9 per 1,000 births and 2.2 per 1,000 births for North America. Abel and Sokol (1991) later reviewed 20 prospective, clinic-based studies (including many of the same studies reviewed in 1987), and reported a lower rate of 0.33 per 1,000 for the western world. By 1994 (Abel 1995), a total of 35 prospective, clinic-based studies had been conducted in at least 40 sites in the western world (including the United States [12 studies], the United Kingdom [5 studies], Australia [4 studies], Spain [3 studies], and Canada, Denmark, France, Italy, the Netherlands, Portugal, Sweden, and Switzerland combined [16 studies]). Many of the studies that were performed outside of the United States were carried out among middle class, Caucasian subjects. Only three of the foreign studies reported any FAS cases, and these three reported only four cases total, producing a very low overall average rate, and median and modal rates of FAS per study of zero. Abel (1995) concluded that FAS occurred “considerably more often” at some sites than at others, estimating the rate for the western world at 0.97 per 1,000 and the rate for the United States at 1.95 per 1,000.^4^ This is a fascinating finding, as the proportion of the population that drinks alcohol in other parts of the western world and the per capita consumption of alcohol is much higher than in the United States. This has been referred to as the “American Paradox” since it is likely linked to the fact that the United States has both more abstainers and more heavy drinkers compared with France and many other parts of the western world (Abel 1998*a*, 1998*b*). Therefore, it is not the prevalence of all drinkers or the amounts that they drink over a long period of time in European countries, but rather the proportion of drinkers who consume substantially large quantities in a short time period that elevates the frequency of occurrence of the major and most severe FAS symptoms, which make up the diagnosis of FAS.

All but four of the U.S. studies (67 percent) reviewed by Abel (1995) were carried out in general obstetric clinic settings among populations that consisted mostly of African-Americans from inner cities who had low socioeconomic status (SES). He concluded that in the United States, sites where the study population was predominantly of low SES and African-American or American Indian, the FAS rate was 2.29 per 1,000, almost 10 times higher than the rate reported for those study sites that were predominantly Caucasian and middle to upper class (0.26 per 1,000) (see table 1). In two of the studies of inner city, low SES populations, the rates of FAS were 3.0 and 3.9 per 1,000. Therefore, Abel’s review concluded that FAS was linked to low SES more than to race. Calculations based on data from another aggregation of these studies (28 studies) indicated that FAS occurred in 4.3 percent of the births to heavy drinkers (defined variously in the different studies) (Abel 1995). Many of the children born to these mothers who are not identified as having FAS would, however, be expected to suffer from conditions related to ARBD and ARND.

Other research projects used prospective, clinic-based studies to document all levels of alcohol-related anomalies (from severe to mild) present in cohorts of children born to mothers who were initially recruited when they were receiving prenatal and other obstetric care. At study sites in Seattle (Streissguth et al. 1990, 1994), Detroit (Jacobson et al. 1993, 1994), and Pittsburgh (Day et al. 1991, 1999), researchers have followed a large number of children born to women with varied levels of drinking and other drug use. They have periodically compared the children on measures of physical growth and development, dysmorphology, and psychological development over time (e.g., 1 to 15 years of age). By grouping the data in various ways to describe the lesser effects of prenatal exposure to alcohol (e.g., reporting on the prevalence of individual symptoms or on the prevalence of cases of FAE, ARBD, and ARND) and documenting the link between the symptoms and alcohol exposure, the researchers provide estimates of the prevalence of these partial FAS cases. One longitudinal study from this body of literature (Sampson et al. 1997) estimated that the combined rate of FAS and ARND is at least 9.1 per 1,000 live births (1 percent) in the general obstetric population of Seattle.

Some studies in other countries in the western world have found a similar pattern of symptoms linked to prenatal alcohol consumption as that found in American studies of ARBD and ARND. For example, French researchers (Rostand et al. 1990) reported that craniofacial morphology was “a sensitive indicator of alcohol exposure in utero” and that “alcoholic” consumption was associated with negative effects on infant weight, length, and head circumference. On the other hand, a study in Australia by Walpole and colleagues (1990) failed to show any significant relationship between low to moderate maternal alcohol intake and fetal outcome. Therefore, in spite of the similar pattern of anomalies associated with low and moderate levels of alcohol consumed during pregnancy, studies outside the United States continue to illustrate the “American Paradox” described by Abel (1998*a*), where low to moderate use of alcohol (defined liberally as less than 21 drinks per week by Rostand and colleagues [1990]) does not result in FAS or symptoms as severe as those reported in U.S. studies.

### Active Case Ascertainment Methods

Active case ascertainment methods were first used among American Indian populations (May et al. 1983). Similar methods have been used in epidemiological studies of more than 24 American Indian and Alaskan and Canadian Native communities (May et al. in press). Until recently, active case ascertainment methods had been used exclusively among American Indians, and primarily among small, well-defined populations. Two recent reports from large community studies using active case finding and ascertainment methods were conducted among first graders in a municipality in South Africa (May et al. 2000), and two counties in Washington State (Clarren et al. 2001).

Active case ascertainment generally yields the highest number of cases and rates of FAS for a particular population. Although the same clinical, diagnostic criteria are used as in the clinic-based studies, the difference in prevalence rates is related to the selection of children who are presented to the clinicians and the age at which clinical contact is made. Apparently, many children who have FAS are not seen in clinics where the proper diagnosis of FAS can be made, or at a time when it can be made. For example, Clarren and colleagues (2001) reported that six of seven first graders who were diagnosed with FAS in their study had never before received a FAS diagnosis. Similarly, Little and colleagues (1990) had previously reported that of 40 newborns in a large hospital in Texas who were strong candidates for a FAS diagnosis (i.e., they were born to heavy drinking mothers and had most of the physical features of FAS), 100 percent left the hospital without a FAS diagnosis. Age at examination is therefore a very important consideration in establishing the true prevalence of this disorder.

Most active case ascertainment studies have been conducted among American Indians in very high-risk communities. These communities are generally characterized by low SES and a small but significant proportion of the population frequently binge drinks. Studies of these communities yield FAS rates that are among the highest in the world. Among Plains and Plateau culture tribes in the United States, prevalence rates vary by community, with average FAS rates of 9 per 1,000 among children ages 1 through 4 (see summary in May et al. in press). Among tribes of the southwestern United States, the rates vary over time, between tribes, and between cultural groups (May et al. 1983). The rates of FAS among southwestern Indians (from birth to 14 years) ranged from 0.0 to 26.7 by community over the time period of 1969–1982, with averages ranging from 1.4 and 2.0 to 9.8 by major cultural group (e.g., Navajo, Pueblo, and southwestern Plains, including Apache and Ute) (see table 1). One active case ascertainment study in Canada examined every child (a total of 102) in a Native village characterized by a concentration of heavy drinkers. The FAS rate reported was 120 cases per 1,000 children (Robinson et al. 1987). Findings in such small and unique communities should not be applied to other, larger populations unless there is substantial evidence that many social, cultural, economic, behavioral, and health conditions are similar across the comparison communities.

The two most recently published active case ascertainment studies were not carried out among American Indians. These studies reported the FAS rates among first grade students (average age of 6) in Washington State and South Africa. In one Washington county, the estimated FAS rate was 3.1 per 1,000 (Clarren et al. 2001). The researchers did not describe the specific social conditions in this county, but this rate is quite high compared with most clinic-based study results. The second study (which is not included in table 1 because it was not carried out in an American population) examined the prevalence of FAS in a town in the Western Cape Province of the Republic of South Africa, a population of generally low SES, including both urban and rural residents (May et al. 2000). The FAS rate was 46.4 per 1,000 for the in-school, first grade population (ages 5 to 9). This particular region of South Africa has a long history of wine production and a high prevalence of binge drinking. As a result, these rates should not be considered representative of populations outside of this particular town or region.

Both of these active case ascertainment studies reported high FAS rates. These results may reflect the selection of high-risk communities, but also the fact that the researchers had complete access to a cross section of the population.

## Risk Factors for FAS, ARBD, and ARND

Epidemiological studies of FAS, ARBD, and ARND, as well as studies of alcohol abusing and dependent women, consistently point to the same risk factors. These risk factors represent a variety of conditions that are frequently associated with the birth of a child with FAS or another alcohol-related condition (ARBD or ARND) (Day et al. 1991; Streissguth et al. 1990). Some of these risk variables may increase risk by leading to heavy prenatal drinking (e.g., family traits and early onset of drinking). Some risk factors are the consequences of alcohol abuse or dependence (e.g., transience, unemployment, and premature mortality). Other variables are biological factors that increase the risk for FAS when associated with heavy drinking (e.g., advanced maternal age and a number of previous pregnancies [i.e., high parity]). In addition, some risk factors are merely associated or correlated with mothers’ lifestyles (e.g., smoking and adverse social conditions), or psychological profiles (e.g., depression, hostility, etc.). Table 2 lists these factors.

Maternal health variables are among the most common and consistent risk factors for FAS and related problems. Many studies, especially those conducted in the United States and Canada, have identified the following health-related risk factors: advancing age of the mother (greater than 25 years of age), having had three or more children, co-use of tobacco and other drugs, and morbidity and premature mortality from alcohol-related causes (NIAAA 2000).

Low SES, as highlighted above, leads the list of social variables that have been found to be associated with FAS births, both in the United States and in other parts of the world (see Abel 1998*b*, 1995 for reviews). But transience and low and marginal employment are also associated with FAS.

Women who give birth to FAS and ARBD or ARND children generally practice frequent and protracted binge drinking, which produces high blood alcohol concentrations. Although a majority of women in many studies reduce their alcohol consumption during pregnancy, mothers of FAS children generally do not (see Abel 1998b; May et al. 2000). Most women who give birth to FAS children live within a social and cultural milieu that tolerates, condones, or is ineffective in dealing with problem drinking. For example, such women often have parents, siblings, and friends who are problem drinkers. Mothers of FAS children often associate and live with men who are heavy drinkers. It is very common for FAS children and their siblings to be in foster or adoptive placement, because high-risk mothers and their families are frequently unable to care for their children adequately and because mothers of FAS children are at increased risk for premature mortality due to their drinking and lifestyle (see May et al. 1983; Clarren et al. 2001).

A few studies have examined the psychological characteristics associated with risky drinking among women and having FAS children. These studies generally find that women in these circumstances suffer from low self-esteem, are depressed, and report problems of sexual dysfunction (e.g., lack of sexual interest, lifetime lack of orgasm or unreliable orgasm) (see Wilsnack et al. 1991; Stratton et al. 1996).

## A Summary of Prevalence Rates

From the review of studies presented here, it is obvious that the lowest rates of FAS are consistently found with passive surveillance methods and that the highest rates are found by the clinic-based and active case ascertainment methods, especially when carried out in high-risk populations. The passive surveillance studies that have produced comparable rates to the studies using other methods either used hybrid methods or studied high-risk populations. For example, a study by Egeland and colleagues (1998) used multiple sources of records and benefited from clinic-based data from birth defect registries and an active case ascertainment project ongoing among Alaska Natives during the period studied. Thus, the more aggressive methods helped to locate more cases than would have otherwise been possible with a standard passive surveillance study utilizing only a centralized reporting system such as birth certificates.

Considering all of the strengths and limitations of the various methods reviewed and the various prevalence findings produced by each method, a simple averaging (calculating a mean) of the results would obviously not produce accurate or reliable estimates for the prevalence of FAS in the general population. Rather, researchers must carefully measure and weigh many details about the demographic and social characteristics of each population to estimate how representative each finding is to the general population. However, based on the average findings of the clinic-based studies and the specific findings of active case ascertainment studies, we find the range of FAS rates to be 0.26 to 2.29 per 1,000 births for the former and 1.4 to 9.8 for the latter. Keeping in mind that active case ascertainment studies generally involve high-risk groups (low SES and high parity, and reservation-based populations of American Indians), and that quite a few of the clinic-based studies in the United States also examined high-risk populations (low SES, inner city populations of high parity), an estimate from these studies alone may be biased on the high side. Therefore, we conclude from this literature review that the overall prevalence of FAS in the United States is likely to be between 0.5 and 2.0 per 1,000 births. Furthermore, many children with ARBD and ARND are born to mothers who drink heavily. Data reported by Abel (1995; 1998*a*) and Sampson and colleagues (1997) provide a guide for us to estimate the rate of all measurable effects of prenatal alcohol exposure. FAS, ARBD, and ARND may affect 10 per 1,000 births (or 1 percent) or more, depending on the specific diagnostic methods and criteria used.

## Conclusion

The epidemiological methods used to study the prevalence and characteristics of FAS, ARBD, and ARND have progressed substantially since the initial studies in the mid-1970s. Useful findings about the prevalence, patterns, and characteristics of risk factors for FAS have improved substantially, leading to the development of a general profile of those mothers who are most likely to have a child with prenatal alcohol damage. The overall prevalence of FAS in the United States was estimated by the Institute of Medicine to be between 0.5 and 3.0 per 1,000 births. Based on the review presented here, which has the advantage of more recent studies, we believe that FAS prevalence in the general population of the U.S. can now be estimated to be between 0.5 and 2 per 1,000 births, and the prevalence of FAS and ARBD combined is likely to be at least 10 per 1,000, or 1 percent of all births. This rate is too high for any population to accept.

Academic and public health professionals, therefore, need to continue population- and clinic-based studies that will further establish the true prevalence of FAS within subsets of the U.S. population and further define the specific behavioral and medical risk factors for prenatal alcohol damage in offspring. The greatest challenges will be to develop better diagnostic and categorical criteria and to conduct further studies of the prevalence and characteristics of ARBD and ARND (Dehane et al. 1991; Stratton et al. 1996). But equally important is the need to use our current epidemiological understanding of FAS and other alcohol-related prenatal problems to design and implement more effective prevention and intervention programs that address these issues across the spectrum of possible public health approaches (see Stratton et al. 1996). Universal prevention approaches can be used to protect entire populations through education and the implementation of public health policy. Selective measures and programs, such as screening for problem drinking during pregnancy and publishing prevention messages, may be designed and aimed at high-risk groups. Finally, interventions such as treatment for alcohol abuse, birth control, and case management can be provided for those women and children who have already experienced the problems of FAS, ARBD, and ARND (see also NIAAA 2000). Current research findings can be used to design a variety of prevention programs to monitor and lower the prevalence of FAS effectively, both in the U.S. and in other countries.

## Figures and Tables

**Table 1:** Summary of FAS Prevalence in Various U.S. and Selected Foreign Studies by Methodology

Type and Location of Study	Years Covered	Rate of FAS per 1,000 births	Population	Source
**Passive Method**				
United States	1979–1992	0.20	General	CDC 1993
	1992	0.37	General	CDC 1995
	1993	0.67	General	CDC 1995
	1981–1986	0.60	African-American	Chavez et al. 1988
		0.08	Hispanic	Chavez et al. 1988
		2.90	American Indian	Chavez et al. 1988
		0.03	Asian	Chavez et al. 1988
		0.09	White	Chavez et al. 1988
Atlanta	1981–1989	0.10	General	CDC 1997
Alaska	1977–1992	0.20–0.30	Alaska, non-native	Egeland et al. 1998
		3.00–5.20	Alaska Native	Egeland et al. 1998
North Dakota	1991–1994	1.10–2.00	General	Burd et al. 1996
**Clinic-Based Studies**				
United States	Various studies (avg.)	1.90	Western World	Abel and Sokol 1987
	Various studies (avg.)	2.20	United States and Canada	Abel and Sokol 1987
	Various studies (avg.)	0.33	United States	Abel and Sokol 1991
United States and other countries	Various studies (avg.)	0.97	Western World	Abel 1995
	Various studies (avg.)	1.95	United States	Abel 1995
	Various studies (avg.)	2.29	African-American	Abel 1995
	Various studies (avg.)	0.26	White American	Abel 1995
**Active Case Ascertainment**				
United States	Various studies (avg.)	9.00	Plains Indian	May et al. in press
	1969–1982	1.40	Navajo	May et al. 1983
	1969–1982	2.00	Pueblo	May et al. 1983
	1969–1982	9.80	Southwestern Plains Indian	May et al. 1983
	1969–1982 (avg.)	1.80	Southwestern Indian	May et al. 1983
Washington State	1995–1997	3.10	1st grade students (one county)	Clarren et al. 2001

NOTE: avg. = average; CDC = Centers for Disease Control and Prevention; FAS = fetal alcohol syndrome.

**Table 2:** Common Risk Factors Associated With Heavy Maternal Drinking, FAS, and ARBD/ARND

Influential Element	Maternal Risk Factor
Health	Older than age 25 when FAS child is born
	Already has three or more children when FAS child is born
	Use of other drugs, including tobacco and illicit substances
	Morbidity or premature mortality from alcohol-related causes
Socioeconomic status (SES)	Low SES
	Social transience
	Unemployment or marginal employment
Drinking pattern	Early age at onset of regular drinking
	Frequent binge drinking (i.e., consuming five or more drinks per occasion 2 or more days per week)
	Frequent drinking (i.e., every day or every weekend)
	High blood alcohol concentration
	No reduction in drinking during pregnancy
Psychological profile	Low self-esteem
	Depression
	Sexual dysfunction
Family social traits	Alcohol misuse in family
	Alcohol misuse by the woman’s male partner
	Tenuous marital status (i.e., cohabitation, never married, separated, or divorced)
	Loss of children to foster or adoptive placement
Local culture and community	Relatively tolerant of heavy drinking

ARBD = alcohol-related birth defects; ARND = alcohol-related neurodevelopmental disorder; FAS = fetal alcohol syndrome.

SOURCES: Adapted from NIAAA 2000; see also Stratton et al. 1996; Abel 1998*b*.

## Footnotes

1 See the sidebar on page 160 for the definition of “prevalence” as it is used in this article.

2 FAS is a set of birth defects caused by maternal consumption of alcohol during pregnancy. It is associated with growth deficiencies and a characteristic set of minor facial traits that tend to become more normal as the child matures. FAS is considered the most common nonhereditary cause of mental retardation. In addition to deficits in general intellectual functioning, individuals with FAS often demonstrate difficulties with learning, memory, attention, and problem solving as well as problems with mental health and social interactions. Children with ARND lack the characteristic facial defects and growth deficiency of FAS but still have alcohol-induced mental impairments. ARBD refers to alcohol-related physical abnormalities of the skeleton and certain organ systems that occur among prenatally exposed children without FAS facial features.

3 Rates of birth defects, including FAS, are usually calculated as the number of children with the disorder expressed as a proportion of each 1,000 live births. Therefore, a rate of one per 1,000 would mean that, on average, one FAS child is born among each 1,000 babies in the given geopolitical area (e.g., the United States). In the United States in 1998, there were 3,941,553 births. Therefore, a rate of FAS of 1 per 1,000 would translate to the birth of 3,942 infants with FAS in that year. Similarly a rate of 0.2 per 1,000 would mean 788 babies with FAS would have been born in the United States in 1998, and a rate of 1.95 per 1,000 would translate to 7,687 infants with FAS in that year.

4 Because U.S. researchers tend to study FAS in high-risk populations and European researchers tend to study lower-risk populations, the rate of 0.97 per 1,000 for the western world might be considered a balanced estimate that is close to the actual prevalence for the United States.
